# Cerebral Organoids as an Experimental Platform for Human Neurogenomics

**DOI:** 10.3390/cells11182803

**Published:** 2022-09-08

**Authors:** Tomasz J. Nowakowski, Sofie R. Salama

**Affiliations:** 1 Department of Neurological Surgery, University of California San Francisco, San Francisco, CA 94158, USA; 2 Department of Anatomy, University of California San Francisco, San Francisco, CA 94158, USA; 3 Department of Psychiatry and Behavioral Sciences, University of California San Francisco, San Francisco, CA 94158, USA; 4 Weill Institute for Neurosciences, University of California San Francisco, San Francisco, CA 94158, USA; 5 Eli and Edythe Broad Center for Regeneration Medicine and Stem Cell Research, University of California San Francisco, San Francisco, CA 94158, USA; 6 Department of Molecular, Cellular and Developmental Biology, University of California Santa Cruz, Santa Cruz, CA 95060, USA; 7 UC Santa Cruz Genomics Institute, University of California Santa Cruz, Santa Cruz, CA 95060, USA

**Keywords:** organoid

## Abstract

The cerebral cortex forms early in development according to a series of heritable neurodevelopmental instructions. Despite deep evolutionary conservation of the cerebral cortex and its foundational six-layered architecture, significant variations in cortical size and folding can be found across mammals, including a disproportionate expansion of the prefrontal cortex in humans. Yet our mechanistic understanding of neurodevelopmental processes is derived overwhelmingly from rodent models, which fail to capture many human-enriched features of cortical development. With the advent of pluripotent stem cells and technologies for differentiating three-dimensional cultures of neural tissue in vitro, cerebral organoids have emerged as an experimental platform that recapitulates several hallmarks of human brain development. In this review, we discuss the merits and limitations of cerebral organoids as experimental models of the developing human brain. We highlight innovations in technology development that seek to increase its fidelity to brain development in vivo and discuss recent efforts to use cerebral organoids to study regeneration and brain evolution as well as to develop neurological and neuropsychiatric disease models.

## 1. Pluripotent Stem Cells

**Figure 1:**
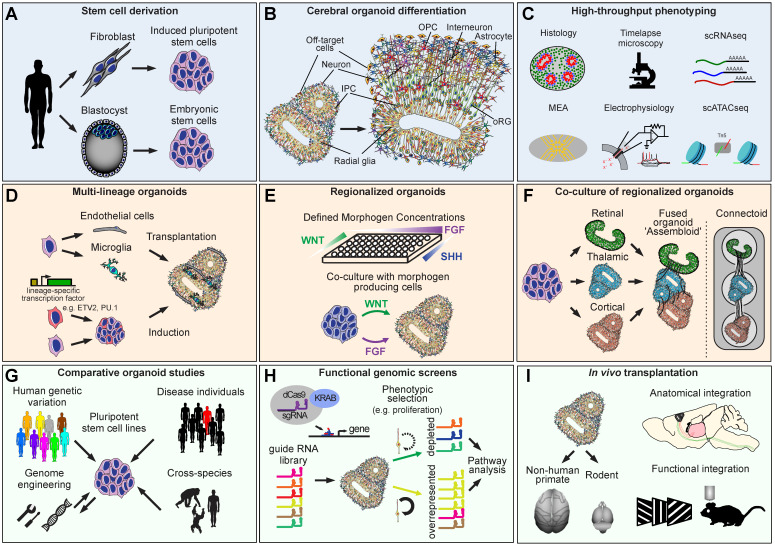
**Derivation, refinement, and applications of cerebral organoid technologies.** (**A**) Pluripotent stem cells can be derived from embryonic material or somatic cells. (**B**) Cerebral organoids recapitulate key cell types of the developing brain. (**C**) Scalable organoid characterization technologies. (**D**) Multi-lineage organoids can be derived to recapitulate tissue cell type diversity more completely. (**E**) Improved regional specificity can be achieved by manipulating developmental signaling pathways according to the blueprint of normal developing tissue. (**F**) Interactions between brain regions can be modeled using co-cultured organoids. (**G**) Organoids derived from different sources of pluripotent stem cells can be used to compare developmental trajectories across individuals, species, or disease states. (**H**) Functional genomic screens can be used to map the genetic architecture of biological phenotypes relevant to neural development. (**I**) Combining organoids with animal models to advance future regenerative medicine applications.

Human tissues are frequently inaccessible to experimentation. In particular, long-term perturbational studies involving human cells resort to cell-based models. This is especially challenging for organs such as the brain, where limited or no stem cell populations can be robustly isolated and cultured in vitro. The isolation of mouse embryonic stem (ES) cells [1,2], the discovery of factors that enable propagation of undifferentiated stem cells in vitro [3], and the establishment of early protocols for neuronal differentiation [4] laid the scientific groundwork for utilizing stem cell-based models of nervous system development. Subsequent isolation of ES cell lines from human blastocysts [5], as well as the discovery of transcription factors that enable reprogramming of somatic cells to ‘induced’ pluripotent stem (iPS) cells [6,7], represent transformative advances that opened up immense opportunities for applying the approach to human cells (Figure 1a). Among early studies, optimized protocols for deriving human cortical cell lineage from embryonic stem cells using small molecules [8,9] and from induced pluripotent stem cells [10] have been instrumental in widespread adoption of the technology by the wider scientific community.

## 2. Characterization

In parallel with the advances in stem cell differentiation into neuroectoderm in serum-free conditions using defined combinations of small molecules, a protocol for differentiating organoids as models of cortical development was introduced [11]. By culturing differentiating cells in three-dimensional aggregates, as opposed to two-dimensional cultures, neuroepithelial stem cells induced from pluripotent stem cells were allowed to self-organize and form clusters (rosettes), forming apical-like structures composed of tight and adherens junctions as well as apical cilia. These apical zone-like structures resemble the early developing cortical neuroepithelium with apico-basal polarity. Glutamatergic neurons emerging from these progenitors were capable of radial migration and formed axonal projections consistent with glutamatergic neuron identities after transplantation into immunocompromised mice [11]. 

The advent of human ES and iPS cells enabled the adoption of these three-dimensional culture protocols for human cells [12,13,14] (Figure 1b). In particular, the pioneering work of Lancaster et al. used a minimally guided differentiation strategy to generate cerebral organoid cultures that recapitulated many distinct brain regions, including dorsal cerebral cortex, ganglionic eminences (ventral cortex), midbrain, and hindbrain, within a single organoid [12]. In contrast to mouse ES cell-derived organoids [11], human stem cell-derived organoids developed a substantial population of outer radial glia (oRG) cells, which are a distinguishing feature of the mouse and human developing brains (discussed in the following section). In parallel, human ES cell differentiation using directed differentiation protocols demonstrated efficient induction of organoids containing only the dorsal cortical neural lineage [14].

Protocols for differentiating cortical organoids have been further refined, and cell types that emerge within brain organoids have been further characterized. For example, the emergence of cortical astrocytes has been demonstrated [13,15,16], as well as that of oligodendrocytes [17]. Neurons have been shown to develop functional synapses [13] and to organize into laminar patterns that resemble cortical layers [16].

More recently, by using multi-electrode arrays to measure spontaneous and evoked activity, organoids have been shown to be able to respond to light stimulation [18], and to develop complex oscillatory patterns of network behavior [19,20]. New engineering approaches are urgently needed to advance the capacity for performing longitudinal extracellular recordings from cultured organoids with minimal interference to their normal neurodevelopmental processes, and exciting solutions are currently being developed [21,22,23]. Integration of such systems with cloud-enabled infrastructure for data processing will be necessary to provide scalability of such methods and wide adoption by the scientific community [24].

Together, these studies have demonstrated a remarkable capacity of neural differentiations to self-organize, recapitulating many complex features of the developing brain, including cell type heterogeneity, cellular organization, and even cell-cell interactions necessary to enable complex patterns of neural activity to emerge (Figure 1b,c).

## 3. Benchmarking against Primary Tissue

Transcriptomic and epigenomic datasets generated from primary human tissue serve as an invaluable resource for benchmarking in vitro models. For example, brain organoids have been profiled using single cell transcriptomics and compared against microdissected bulk tissue transcriptomic data from developing human cerebral cortex, revealing that the molecular programs underlying human cortical neurogenesis are broadly expressed in cortical organoids [25]. This study also revealed that organoids contained primarily the dorsal telencephalic neural lineage with a small proportion of non-neural cells. Thanks to the generation of multiple single cell transcriptomic datasets from primary human tissue [26,27,28,29,30,31], it is now possible to compare cell types emerging in brain organoids to their primary counterparts in even greater detail. These studies have revealed that as development proceeds, cells from brain organoids fail to accurately recapitulate the full breadth of mature cell types and the associated diversity of specific patterns of gene expression found in primary tissue. Furthermore, organoid cells upregulate molecular signatures of cell stress [32,33]. Similar findings have been reported more broadly for in vitro cultured cells [31], including cultured human primary cells [33]. Recently developed bioinformatic approaches can be leveraged to regress stress signatures [34]. As new protocols and approaches are being developed to limit the effects of cell stress in culture conditions, single-cell transcriptomic studies will serve as an invaluable resource for comparing the fidelity and robustness of these protocols to the normal developing brain. Data sharing resources, such as the UCSC Cell Browser, have emerged to facilitate rapid data sharing and access, particularly for single cell RNA sequencing datasets whose processing steps have been largely standardized [35].

Similarly to transcriptomic analyses, epigenomic and epitranscriptomic (mRNA modifications) profiling have been applied to brain organoid and primary human tissue samples, which also revealed that broad neurodevelopmental trajectories and transitions are recapitulated in organoids sampled at different stages of differentiation [36,37,38,39]. Single cell epigenomic datasets from primary human tissue are only beginning to be generated [40,41]. Therefore systematic comparisons of epigenetic states between primary tissue and organoid cell types remain to be performed. Chromatin accessibility changes occurring during neurodevelopmental transitions have been shown to occur in brain organoids, and organoid-derived neural cells recapitulate a substantial fraction of the putative enhancers identified in developing human tissue [39,41].

**Figure 2:**
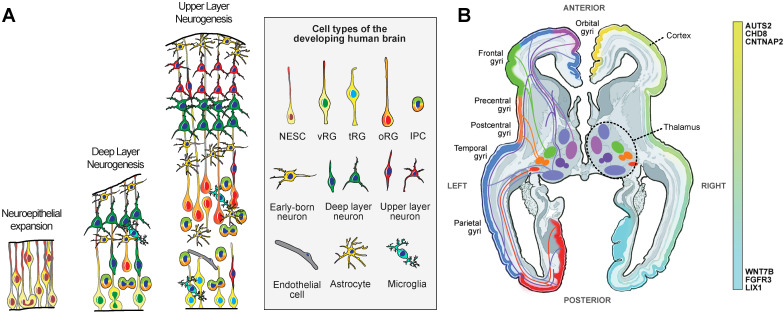
**Development of the human brain.** (**A**) Schematic representation of the key cellular populations during stages of peak neurogenesis and early gliogenesis in the human cerebral cortex. NESC—neuroepithelial stem cells, vRG—ventricular radial glia, tRG—truncated radial glia, oRG—outer radial glia, IPC-intermediate progenitor cells. (**B**) Development of the long-range connectivity between prospective subdivisions of the thalamus and cortical areas. The schematic shows a horizontal section through a developing human brain. The left half highlights major projection pathways between the emerging thalamic nuclei and cortical areas. The right half highlights differences in expression levels of specific genes with rostro-caudal expression gradients.

While transcriptomic and epigenomic data are tremendously valuable in that they enable genome-wide comparisons, they are unable to capture the dynamic neurodevelopmental processes that give rise to the complex cell types of the developing brain. Mechanistic studies of cortical development have been overwhelmingly conducted in mice. These studies have underscored the role of radial glia as the neural stem cells of the brain [42,43,44]. Radial glia are a specialized cell type that consists of a spindle-shaped cell body with bipolar morphology, including an apical fiber contacting the lateral ventricle and forming an apical junction, and a basal fiber extending to and contacting the pial surface [45] (Figure 2a). These cells have been shown to generate glutamatergic neurons directly [46,47] or indirectly via intermediate neural progenitor cells [48,49,50]. In the cerebral cortex, neurogenesis occurs within a specific time window [51] and in a temporally hierarchical order, with deep layer neurons generated before upper layer neurons [52]. During mouse cortical development, neurogenesis is followed by gliogenesis [44,48,53]. This series of neurodevelopmental events is often referenced as a template for cortical development across mammalian species.

In many species outside mice, such as ferrets, macaques, and human developing cerebral cortex, radial glia extensively diversify into outer radial glia (‘oRG’, also referred to as ‘basal’ radial glia) in the outer subventricular zone (OSVZ) [54,55,56,57,58,59], and truncated radial glia [60,61] in the ventricular zone (VZ). At late stages of cortical development, the OSVZ harbors most of the proliferating cells in the developing cerebral cortex [54]. The discovery of molecular programs enriched in oRG cells [62,63] has identified mechanisms by which the development of these cell types can be promoted, and the hypothesis that LIF/STAT3 signaling can enhance oRG cell generation has been tested in organoids [64]. The role of this pathway was surprising, given the central role of this pathway in gliogenesis [65].

Importantly, oRG cells have been shown to give rise to oligodendrocyte progenitor cells (OPC) [66], although new studies suggest that other neural stem cell populations may generate OPCs at early developmental periods [67]. Astrocytes have been shown to be a major cellular output of oRG cells [68,69], but recent studies have revealed further complexity that exists within this cell class in both humans and mice [70,71,72,73], which underscores the need for further studies of their developmental cell lineage. Adding to this complexity, human glial progenitor cells have been shown to generate oligodendrocytes and astrocytes [74], and overlapping molecular signatures of the oligodendrocyte and astrocyte lineages have been identified in single cell studies [40,75,76].

Glutamatergic neurons of the cerebral cortex are generated by radial glia and IPCs during human development [54,55,56,57,58]. At late stages of cortical neurogenesis, oRG cells have been shown to generate neurons directly and indirectly [55]. Notably, periods of neurogenesis and gliogenesis in humans, as well as ferrets, overlap extensively [60,71], in contrast to mice [44,48,53]. Importantly, glutamatergic neurons that emerge in anatomically distinct areas of the cerebral cortex are molecularly divergent [27,30].

GABAergic neurons of the cerebral cortex originate in the ganglionic eminences [77,78,79,80,81]. However, a substantial fraction of progenitor cells and newly born neurons in the cerebral cortical wall at late stages of neurogenesis express markers of GABAergic lineage [28,82,83,84]. This presumed local production of cortical GABAergic neurons has been confirmed by a barcoded lineage tracing study of human cortical radial glia [79]. Notably, many studies utilizing cortical organoids have observed GABAergic neurons emerging at late stages of differentiation, but their origin and subtype identity remain unclear [32,85,86].

A deeper understanding of radial glia heterogeneity and differentiation trajectories in the human cerebral cortex will provide critical resources for benchmarking cerebral organoids as models of human neural development and may provide important insights into the cell of origin of brain tumors [87,88,89]. While many bioinformatic methods have been developed to reconstruct developmental trajectories from single cell transcriptomic data [90,91], they are limited in several important ways that may be important for our understanding of developmental processes [92]. Timelapse microscopy experiments in human and non-human primate tissue have provided critical insights into the complex patterns of progenitor cell behavior and differentiation trajectories in the developing brain [55,56,93,94]. These studies have revealed the neural stem cell potential of oRG cells [56], demonstrated evolutionary conservation of oRG cell behaviors [93], identified mechanisms underlying oRG cell generation [94], and revealed the remarkable neurogenic capacity of oRG cells, including their striking potential to give rise to neurons via direct neurogenesis in addition to their capacity to generate intermediate neural progenitors (‘indirect neurogenesis’) [55,62].

Timelapse microscopy studies conducted in cerebral organoids have demonstrated that the characteristic behaviors of radial glia subtypes are recapitulated in organoids [95,96], as well as their capacity to differentiate via direct and indirect neurogenesis [97]. In addition to timelapse microscopy, technologies that enable multiplexed tracking of cell lineage at high-throughput using single cell transcriptomics can be applied to cerebral organoids [98,99] and primary tissue [79,100,101]. Examining mechanisms underlying human brain development using brain organoids will critically depend on rigorous benchmarking of the differentiation trajectories of neural progenitor cells.

## 4. Protocol and Data Standards

Cerebral organoid protocols are being widely adopted to model human neurodevelopment, the consequences of genetic mutations, and in the context of evolutionary comparisons. In parallel, advanced methods are being developed to apply next-generation technologies to brain organoid culture. Publications describing detailed protocols as well as hands-on training workshops represent important steps towards developing protocol commons, as well as quality control standards that could be agreed upon by the scientific community to support the generation of interpretable data. One difference in experimental design that could lead to different experimental outcomes involves the choice of neural induction protocol. Some studies have relied on protocols that are more directed and involve dual smad inhibition [11,13,14], while other studies utilize minimally guided differentiations, which result in the generation of multiple lineages within the same organoid [12]. Depending on the nature of the assay (e.g., single cell transcriptomics or neural activity), the results of studies utilizing these divergent methods could require different interpretative frameworks.

Another important issue that is rarely discussed is the fact that lines from very few donors have thus far been utilized by most organoid studies. There is an unmet need to establish lines from donors that represent a more complete spectrum of human genetic diversity. Validation of their differentiation capacity using newly developed protocols that increase the reproducibility of cerebral organoid differentiations [85,102] will be key to advancing the field of organoid research. Equally important, datasets generated from cerebral organoids should ideally follow the principles of findability, accessibility, interoperability, and reusability (FAIR standards) [103]. Such datasets, together with platforms for data sharing, will be necessary to enable rigorous comparisons of experimental results from studies involving organoids. It is not unexpected that protocols and outcomes will vary depending on the research question being asked and the cell lines being used. However, benchmarking the data generated in cerebral organoid studies to both human primary tissue compendia and publically available cerebral organoid datasets, such as those in the UCSC Cell Browser, will go a long way towards establishing the relevance and utility of newly published cerebral organoid studies.

## 5. Reducing Stress

A pervasive cell culture-associated cell stress response has been identified as a potential confounding factor in organoid experiments [32,33]. Some of the potential consequences include genomic instability and the acquisition of somatic mutations in iPS derived models [104]. To overcome these limitations, algorithmic approaches have been developed to regress gene expression signatures related to cell stress [34]. While a variety of approaches are being examined, protocols that incorporate slicing of organoids or culture at the air-liquid interface [16,105] have shown a substantial reduction in the level of hypoxia within organoids. Moreover, incorporation of non-neuronal cells that do not emerge spontaneously in large numbers during neural differentiation may provide beneficial outcomes for organoid development. For example, microglia are the tissue-resident macrophages of the brain that arise in the yolk sac and are involved in innate immune and homeostatic functions [106]. Incorporation of microglia into organoids via transplantation or induction of the PU.1 transcription factor expression in a subset of organoid cells [107,108,109,110] (Figure 1d) has been shown to attenuate DNA damage responses in organoids and to promote the maturation of neuronal activity.

Similarly, most cell types that form the cerebrovasculature [111] do not arise from neural stem cells but can be introduced into organoids by co-culture [112,113,114] or by ectopic expression of the ETV2 transcription factor [115] (Figure 1d). Endothelial cells can self-organize to form tubular structures reminiscent of early developing vascular networks and can improve the viability of organoid cells and enhance functional maturation [115]. An important future direction involves developing vascular cell differentiation protocols that can more rapidly generate vascular cells at high purity [116], as well as those that more robustly resemble cerebrovascular endothelial cells as opposed to endothelial cells found in other organs [117].

Other cell types that are involved in cerebrovascular interactions, such as pericytes, smooth muscle cells, fibroblasts, fibromyocytes, or perivascular macrophages [111], remain to be explored. Incorporation of flow may be required to support successful vascularization of brain organoids and enable mechanistic studies of neurovascular interactions and disease modeling using organoids. Dysfunction of the cerebrovascular system is thought to be central to the pathophysiology of vascular malformations and neurodegeneration [118,119]. Therefore developing vascularized organoid models will be important for studies of these conditions using human cell-based models.

## 6. Multi-Brain Region Organoids

During development, neurons form local and long-range interactions with cells located in ontogenetically distinct regions (Figure 1e). While the initial differentiation protocols focused on accomplishing high efficiency differentiations for dorsal cortex organoids, protocols that enable other brain region specific differentiations have also emerged, including for ganglionic eminence [120,121,122], striatum [123], hippocampus [124], hypothalamus [86,125], pituitary gland [125,126], thalamus [127,128], midbrain [86,129,130,131], and cerebellum [132] organoids. Algorithms that enable data-driven alignment of organoids to a spatially resolved transcriptomic atlas of the developing brain can provide rapid validation of newly optimized differentiation protocols. Specifically, by leveraging spatially resolved gene expression data, such as the Allen Institute RNA in situ hybridization and laser capture microdissection microarray databases, the VoxHunt algorithm can annotate organoid single cell RNAseq data with brain regional identity information [133]. One limitation of this method is that the reference data currently available are not transcriptome-wide or single cell resolved. With the advent of brain region-resolved single cell or spatial datasets with single cell resolution, the accuracy of predicting regional identities of organoid cells will likely increase. Broader comparisons to not only brain tissue but also non-brain reference data will additionally enable fully agnostic, data-driven benchmarking of organoid cells against the blueprint of developing human tissue.

Physical co-culture of two or more brain region-specific organoids enables interactions between cells that emerge from different sets of progenitors to be studied in vitro. This approach is sometimes referred to as an ‘assembloid’ assay (Figure 1f), and allows for new neurodevelopmental processes to be assayed in vitro, such as the migration of GABAergic neurons from ventral telencephalic progenitors to the cortical plate [120,121,122]. Early developmental events involved in the formation of long-range neuronal tracts can also be examined, such as the cortico-striatal [123] or cortico-thalamic tracts [128]. In another exciting application, organoids co-cultured with non-neural tissue, such as muscle tissue [134,135], demonstrated the potential for organoid neurons to functionally innervate non-neural tissues, opening an exciting new frontier for regenerative medicine research (Figure 1g).

However, organoid fusions require parallel organoids to be differentiated into distinct regions, and in most cases, organoid fusion is not performed until these regional identities are fully acquired. As an alternative strategy, a growing number of studies are employing methodologies that deliver localized sources of developmental morphogens into parts of an organoid, such that they can mimic the effects of organizer regions that normally pattern the developing neuroepithelium. As a result, multiple brain regions can be simultaneously induced within the same organoid [136,137].

Another experimental approach that enables studies of neural interactions involves the separate induction of brain organoids that are cultured separately while allowing for neurons to form reciprocal axonal connections. These “connectoid” cultures have been shown to develop more complex neural activity profiles [138] than individually cultured organoids [20] (Figure 1f).

## 7. Specification of Cortical Neuron Subtypes

Cortical organoid differentiation protocols have been convincingly shown to generate cortical lineage neurons. However, the brain contains dozens of neuronal subtypes that ultimately give rise to its complex function. In this section, we will discuss broadly how the diversity of neurons in the mammalian forebrain is specified and how these are modeled using organoids.

Cortical neurons are specified along two major axes. First, their position in the six-layered cortical plate defines their morphology and connectivity. For example, “deep layer” neurons located in layers five and six send axons to subcortical areas, including the spinal cord and thalamus, respectively, whereas “upper layer” neurons of layers two, three, and four project to other cortical layers, to the contralateral hemisphere, or to the striatum [139]. The specification of cortical layer neurons follows a temporal, inside-out, order [52] and involves the induction of transcription factors that specify the major neuronal subtypes [140]. The sequential generation of deep and upper layer neurons is recapitulated in iPS derived cultures and cerebral organoids [8,10,141], although the stereotypical layering of cortical neurons has only been reported in a few studies [16].

The second axis follows the surface of the cerebral cortex, which can be divided into dozens of anatomically and functionally distinct areas, called aerial or regional identities. During early development, developmental morphogens secreted by organizer regions induce the expression of patterning transcription factors in radial glial cells, which subsequently give rise to glutamatergic neurons (reviewed by [142]). Different areas of the cerebral cortex have transcriptionally divergent glutamatergic neurons [27,30] (Figure 2b).

As an early application of the organoid technology, Sasai et al. demonstrated that cortical neuron differentiation can be further refined by harnessing lessons from developmental biology to increase regional specificity. In their experiments, inhibiting FGF signaling favored the specification of caudal cortical identities (NR2F2+ ve neurons), whereas activating Wnt and BMP signaling favored the specification of medial structures such as the cortical hem and choroid plexus [11]. Subsequently, these findings have been extended to form the basis for developing protocols for generating hippocampal organoids [86,124]. By modulating the duration of FGF signaling exposure during initial patterning, Studer et al. developed a protocol that promotes the specification of prefrontal cortical neurons [143].

Regulatory programs underlying cortical neuron specification and arealization have been extensively studied in mice [142]. Targeted knock-down of such master-regulatory transcription factors, such as GLI3, using CRISPR can be used to examine the effects of the manipulation on neuronal subtype specification [99] (Figure 1h), recapitulating the findings from mouse models [144].

Finally, epigenomic datasets generated from distinct areas of the cerebral cortex are beginning to emerge [41,145,146], and these studies have the potential to uncover regulatory programs underlying cortical arealization more comprehensively. For example, transcription factor motif enrichment analysis of prefrontal cortex enriched open chromatin regions identified retinoic acid receptor motif enrichment. Mechanistic studies of retinoic acid signaling have revealed a role for this pathway in the specification of the prefrontal cortex in the developing human brain and shown that modulation of retinoic acid signaling during organoid differentiation can regulate the differentiation of prefrontal and occipital cortical neurons in cerebral organoids [41,147]. Additional studies are needed to more comprehensively predict the regulatory grammar of cortical arealization which could be leveraged to derive next-generation protocols for generating area-specific cortical organoids.

## 8. Transplantation

One of the key applications of stem cell-based technologies is regenerative medicine. Pluripotent stem cell derived neurons have been successfully transplanted into neonatal immunocompromised mice and shown to develop projection patterns consistent with their molecular identities [11,148]. Moreover, transplanted human cells have been shown to integrate into mouse cortical circuits and develop evoked responses consistent with in situ neurons [149] (Figure 1i). The remarkable capacity of transplanted cells to integrate into the brain offers a promising outlook for future application of this approach in clinical settings. However, most transplantation paradigms are performed in the early postnatal period, when the majority of the mouse brain is still undergoing normal developmental processes. Transplantation into the adult brain is highly inefficient and rarely performed.

Several pioneering studies have successfully transplanted intact organoids into adult mouse brains [150,151,152,153]. These studies have shown that neurons within brain organoids continue to undergo normal developmental transitions, integrate into cortical tissue, and form functional synapses with mouse cells. Endogenous vascular cells of the mouse have been shown to progressively vascularize brain organoids.

In one study, organoids were transplanted into 3-year-old cynomolgus monkeys [152]. By 12 weeks post transplantation, axons of organoid neurons extended projections within the cortex, to the corpus callosum, and the striatum. No projections were found extending through the internal capsule, but this may be unsurprising given that the vast majority of neurons in the grafted organoids expressed SATB2, a marker of intratelencephalic neurons [154]. Future optimizations may be needed to develop protocols that could enhance the survival and integration of other neuronal subtypes into the monkey brain, including corticospinal and corticothalamic projection neurons.

While chimeric animal studies are necessary to understand the fidelity and function of in vitro derived human brain tissue and are a necessary first step for regenerative medicine approaches, they are not without controversy. Biomedical researchers, ethicists, and the public have questioned whether and when transplanting human neural tissue into experimental animals leads to human-like perception or cognition. Would chimeric animals modeling neuropsychiatric diseases potentially experience human disease symptoms in a distressing manner? The National Academies of Science, Engineering, and Medicine published a report on neural chimeric tissues in 2021 [155] that called out areas of concern with respect to this research, although it also stated that current regulation of stem cell and animal research was adequate. This issue continues to spur a lively debate in the research community that will only increase as brain organoids increase in their complexity and similarity to human brain tissue [156].

## 9. Disease Modeling

The discovery of reprogramming factors has allowed for the generation of pluripotent stem cells from patient cells, allowing for in vitro studies in a patient-specific genetic background. As a result, one of the most common applications of iPS technology today is disease modeling. Two early studies differentiated glutamatergic neurons from iPS lines derived from idiopathic schizophrenia [157] and Timothy Syndrome [158] patients. These studies heralded an era of disease modeling using human cell-based models [159]. The results of these studies have been extensively discussed in a number of recent reviews [160], and will not be further discussed in this review.

In addition to patient-derived cells, genome engineering technologies using CRISPR have also enabled the generation of ‘isogenic’ iPS lines where a putative disease-causing variant can be introduced into control iPS lines or corrected in a patient iPS line. This approach offers tremendous potential for screening large numbers of candidate mutations as well as for assessing the role of genetic background in specific disease-associated variants. However, rigorous validation of isogenic iPS lines will be necessary because non-specific ‘passenger’ mutations are frequently introduced during clonal selection of isogenic lines [161].

### 9.1. Neurological Disorders

Cerebral organoids generated from patient-derived iPS lines have also been applied to studies of disease mechanisms. For example, organoids derived from an iPS line derived from a patient with compound heterozygous truncating mutations in CDK5RAP2 have been shown to exhibit slower growth kinetics compared to control iPS lines [12]. This study also identified precocious differentiation and alterations in the cell division plane of radial glia that likely underlie the microcephaly phenotype. This study was, to the best of our knowledge, the first application of cerebral organoids to disease modeling.

The advent of new technologies, such as single cell barcoding and CRISPR perturbation screening, has enabled parallel and multiplexed perturbations of genes in cerebral organoids. Esk et al. applied this approach to study the effects of loss of function of 173 candidate microcephaly genes [98]. This study has revealed a novel role for genes involved in endoplasmic reticulum (ER) function as a point of vulnerability in microcephaly.

Mechanisms underlying microcephaly have been successfully studied using mouse models thanks to the conservation of many biochemical processes of cell division. By contrast, lissencephaly is a condition inherently difficult to model in mice due to the lissencephalic nature of the mouse cerebral cortex. LIS1 (encoded by *PAFAH1B1*) encodes one of the five genes implicated in primary lissencephaly in humans [162]. Studies in mice have found subtle neurodevelopmental phenotypes in transgenic mice with Lis1 loss of function, including neuronal migration and neurite extension deficits [163,164]. By contrast, introduction of LIS1 loss of function mutations into human ES cell lines using CRISPR, followed by differentiation into cerebral organoids, was sufficient to detect a striking difference in neuroepithelial stem cell and radial glia proliferation [165], suggesting that, in humans, LIS1 could play an important role beyond the control of neuronal migration that was discovered using a mouse model.

Miller-Dieker Syndrome is characterized by lissencephaly but also microcephaly, and organoids derived from patients with this condition show a number of distinct phenotypes impacting the survival of neuroepithelial cells, outer radial glia cell division, and neuronal migration [95]. Notably, Miller-Dieker Syndrome patients carry mutations in *PAFAH1B1* in addition to other genes, highlighting the need for isogenic lines for dissecting the contribution of individual genes to distinct phenotypes. 

Tuberous sclerosis is a rare condition that involves the formation of many non-cancerous tumors throughout the body. Tuberous sclerosis patients often develop epileptic seizures [166], and two high-confidence risk genes, *TSC1* and *TSC2*, have been identified [167,168]. Both TSC1 and TSC2 act as negative regulators of the mammalian target of rapamycin gene (mTOR) in mammalian cells [169]. In the brain, mTOR signaling has been shown to be a major regulator of synaptic function, and its inhibition has been shown to rescue synaptic phenotypes in tuberous sclerosis [170]. However, mTOR signaling also plays a significant role in brain development. In humans, mTOR signaling is highly enriched in oRG cells [27] and regulates radial glia fiber maintenance [171]. By contrast, the radial glia of the developing mouse brain are less dependent on mTOR signaling [172] and do not develop tubers, arguing for the development of a human cell-based model of tuberous sclerosis. Organoids derived from human ES cell lines with *TCS1* and *TSC2* mutations have been shown to have precocious gliogenesis that can be rescued by rapamycin inhibition [173].

Another study used cerebral organoids to investigate neurodevelopmental phenotypes underlying periventricular heterotopia by generating mutations in *DCHS1* and *FAT4*. Radial glia deficient for these proteins showed severe disorganization of radial morphology and neuronal migration defects, as well as possible defects in neuronal fate specification [174].

### 9.2. Psychiatric Conditions

Perhaps the most aspirational application of brain organoids is in studies of psychiatric disorders, where iPS-derived organoids allow researchers to study human neurodevelopmental processes in the human genetic background. This may be especially important in conditions such as schizophrenia or autism spectrum disorders (ASD), which involve the contribution of many genetic variants. Polygenic risk architecture may be difficult to capture in animal models.

Starting from syndromic disorders that include nervous system alterations, iPS cells derived from patients with Timothy syndrome have been used to identify neural deficits associated with the condition [158], including calcium signaling defects in progenitors and neurons. More recently, assembloids derived from cortical and ganglionic eminence organoids have been used to show that tangentially migrating interneurons exhibit abnormal patterns of neuronal migration in Timothy syndrome and can be rescued by inhibiting GABA-A receptor signaling [120,175].

Several studies utilized pluripotent stem cells derived from patients with DiGeorge syndrome, which is caused by a microdeletion at the 22q11.2 locus. Cortical differentiations of these lines have been shown to result in precocious gliogenesis [176] and a delayed switch of the GABA reversal potential [177]. In addition, mitochondrial defects have been identified [178]. Cortical organoids derived from 22q11.2 microdeletion patients have been shown to exhibit neuronal hyperexcitability that could be rescued by restoring the expression of DGCR8 [179], an enzyme critical to the synthesis of microRNAs [180].

MECP2 encodes a CpG binding protein and is mutated in patients with Rett syndrome [181,182]. Rett syndrome patient-derived iPS cells differentiated into cortical glutamatergic neurons have been shown to develop reduced synaptic arbors, a phenotype that could be rescued by overexpression of MECP2 as well as the addition of IGF1 based on findings from a mouse model [183,184]. Inhibition of ribosomal proofreading activity using gentimicin was also suggested to elevate MeCP2 expression, but the effect was highly sensitive to drug concentration [184]. A subsequent study using brain organoids further revealed neuronal network activity phenotypes that could be attenuated using Nefiracetam and PHA 543,613 [185]. Fused cortical and ganglion eminence organoids derived from stem cell lines carrying loss of function mutations in *MECP2* have been shown to exhibit abnormal patterns of oscillatory network activity that can be partially rescued by Pifithrin-α, a TP53 inhibitor [186], consistent with studies in patient derived fibroblasts showing increased induction of P53 and senescence [187]. These studies illustrate the complexity of neurodevelopmental processes regulated by MeCP2 and suggest that MeCP2 loss of function consequences may be mediated by many biological processes and do not converge upon a small number of biochemical targets.

Studies of ASD pathobiology have attracted substantial interest in the scientific community due to the complex nature of this early-onset condition. Human genetics studies have implicated hundreds of genetic variants that might underlie ASD [188,189,190]. The genetic architecture of ASD involves rare as well as common variants and following the discovery of high-confidence ASD risk loci comes the challenge of understanding their function.

Copy number variants in the 16p11.2 locus are strongly associated with ASD diagnosis [191,192]. Studies investigating neurodevelopmental phenotypes using two-dimensional cultures of patient-derived cells identified several phenotypes associated with 16p11.2 microdeletion [193]. Neurons derived from cells with 16p11.2 deletion showed enlarged cell soma and deficits in synaptic morphology. In addition, a study of cortical organoids derived from 16p11.2 microdeletion patients revealed deficits in neuronal migration, and deficits in Wnt signaling pathway activation, and a reduced pool of neural progenitor cells [194]. Additionally, a recent study measuring gene expression in 16p11.2 microdeletion organoids derived from 13 donors implicates neural progenitor cells in early developmental changes and suggests transcriptional dysregulation through gene coexpression network analysis [195].

High-confidence ASD risk genes discovered through whole exome sequencing studies coalesce into several categories, including gene expression regulation and synaptic transmission. Understanding the points of convergence between them that might underlie the highly stereotypical nature of ASD symptoms remains challenging. However, early analyses have uncovered brain regions and developmental time points that are enriched for ASD risk gene expression. Specifically, by intersecting ASD candidate risk genes with gene expression information from the developing human brain [196], two studies identified enriched expression of ASD risk genes in prenatally developing human prefrontal cortex [197,198]. Intersection of these genes with single cell expression data from the human brain [27,29] has further shown that many ASD risk genes show enriched expression in glutamatergic and gabaergic neurons [188,190]. Classical annotations of protein function are often based on studies in cell lines and animal models. This can inaccurately capture the complex roles of these proteins across the wide spectrum of cells in the human brain. Moreover, assumptions that expression level correlates with functional significance can be misleading. For example, the vast majority of studies into the role of the ASD risk gene SynGAP1 have been focused on the role of this protein in synaptic transmission [199]. Indeed, in the developing human brain, *SYNGAP1* mRNA is enriched in glutamatergic neurons, but its expression can also be detected in radial glia [27]. Emerging evidence from cerebral organoids suggests that SynGAP1 may also play a role in radial glia during early neurogenesis [200]. This finding highlights the need to systematically interrogate gene function across the range of cell types where risk genes are expressed.

In an effort to systematically screen high-confidence ASD risk genes for their function in neurodevelopment, several studies have begun to interrogate neurodevelopmental phenotypes for multiple genes in parallel, using iPS-derived neurons and cerebral organoids.

Cederquist et al. generated isogenic pluripotent stem cell lines carrying loss of function mutations for 27 high confidence ASD risk genes and differentiated the cells in a pooled assay to glutamatergic neurons of the prefrontal cortex [143]. Loss of function mutations in CUL3, KDM5B, ASH1L, ASXL3, ANKRD11, RELN, DEAF1, and KMT2C resulted in decreased neurogenesis. Mutations in KMT2A, SUV420H1, DYRK1A, GRIN2B, and CHD8 resulted in proportionately increased numbers of newborn neurons at the expense of progenitors.

Lalli et al. investigated progenitor cell proliferation and neuronal differentiation phenotypes across 13 high-confidence ASD risk genes using a pooled CRISPR screen approach in neural progenitor cells [201]. This study found that out of the 13 ASD risk genes investigated, knock-down of five genes (CHD2, ARID1B, ADNP, ASH1L, and DYRK1A) resulted in reduced proliferation, while knock-down of two genes (PTEN and CHD8) led to accelerated maturation and increased proliferation. Four genes (ASH1L, ADNP, ARID1B, and DYRK1A) resulted in reduced neurite extension, while one gene (PTEN) led to increased neurite extension in cortical neurons.

An organoid-based study investigated the neurodevelopmental consequences of *SUV420H1* (also known as *KMT5B*), *ARID1B*, and *CHD8* loss of function using comprehensive single cell RNA sequencing analysis. In this study, heterochrony of neuronal maturation and excessive production of GABAergic versus glutamatergic neurons were observed [202]. Transcriptional changes between mutant and control cells were found to be more consistent across cell types within candidate mutations (especially for ARID1B and CHD8) than across candidate genes. A parallel study using CHD8 isogenic mutant cerebral organoids reported alterations in the proliferative capacity of progenitor cells, with prolonged proliferation of mutant cells [203].

Together, these studies suggest that loss of function mutations of at least a subset of high-confidence ASD risk genes disrupt radial glia proliferation or neuronal differentiation. This is consistent with clinical reports suggesting that patients with ASD can have either increased or decreased head circumference relative to the general population [204]. However, while functional genomic information may support patient stratification, the results of these studies do not offer a simple answer to the question of phenotype convergence across diverse risk genes.

Complementing the studies of rare risk variants, organoids derived from idiopathic ASD patients have identified phenotypes of increased abundance of cortical interneurons [205,206] in addition to alterations of cortical progenitor cell proliferation. These findings appear to be consistent with the recent study in organoids with SUV420H1, ARID1B, and CHD8 mutations [202]. Follow-up studies are beginning to further investigate this question using iPS lines derived from idiopathic ASD individuals who were either normocephalic or macrocephalic [207]. Phenotypic differences in progenitor cell development, neurogenesis, and neuronal differentiation between donors stratified according to macrocephaly status may provide important insights into neurodevelopmental phenotypes underlying patient phenotypes.

Additional studies are needed to investigate the consequences of ASD risk gene mutations on additional neurodevelopmental phenotypes, including cell behavior, epigenetics, neurophysiology, synaptogenesis, cell dynamics, gliogenesis, and connectivity. Progress in addressing these questions will require advances in culture methods that promote neuronal maturation [208,209], scalable methods combining multiplexed perturbation strategies with increasingly complex phenotypes, such as pooled optical CRISPR screens [210], or perturb-ATAC [211]. Moreover, it will be important to apply these strategies across a wide range of cell types, not limited to a specific brain region or even just the neural lineage. The advent of quantitative frameworks that can take advantage of sparse datasets to learn latent representations of biological phenotypes will be critical to interpreting the results of such experiments [212,213].

One of the general themes that has emerged thus far from organoid-based modeling of neurodevelopmental phenotypes associated with psychiatric variants is that such mutations frequently lead to aberrant timing of neurodevelopmental events. Such heterochronicity could lead to disorganized development of neural circuits, likely leading to functional abnormalities. Given the inherent variability of cerebral organoid differentiations as well as the current inability to precisely control many neurodevelopmental processes, such as the positional identity of radial glia, insights gained from organoids should be carefully validated to determine their predictive value.

However, validating early neurodevelopmental phenotypes in human tissue can be challenging due to the limited availability of suitable research material. Still, insights gained from brain organoids can and should lead to predicted consequences in brain structure or function. Functional imaging studies that have been performed in patients with psychiatric conditions can serve as a point of reference [214]. Similarly, studies of postmortem tissue derived from patients with autism or schizophrenia have provided insights into molecular and cellular changes associated with these conditions [215,216,217,218,219]. Even though postmortem tissue likely involves additional changes related to co-morbidities and drug treatments, one would expect some degree of overlap between primary tissue and organoid models, especially where the comparison is performed between samples with the same mutation. Finally, comparisons to animal models can be extremely useful in identifying robust phenotypes and can serve as a guiding principle for the use of such models in preclinical studies [220,221].

### 9.3. Neurodegeneration

Another area of growing interest is the application of organoid technology to interrogate mechanisms underlying neurodegenerative phenotypes. Neurodegenerative disorders involve many genetic and non-genetic risk factors and are thought to involve dysfunction of homeostatic mechanisms involving not only neurons but also non-neuronal cells. Four major disease areas are being actively studied: Parkinson’s disease, Huntington’s disease, Alzheimer’s disease, and Down syndrome.

Multiple studies have leveraged patient derived iPS cells to recapitulate deficits in neuronal survival as well as abnormalities in biochemical processes relevant to these conditions [31,222,223,224,225,226]. For an in-depth review, see [227].

Cerebral organoids have also been applied to the study of neuronal phenotypes associated with neurodegeneration. Organoids derived from patients with familial Alzheimer’s disease as well as Down syndrome have been shown to recapitulate toxic accumulation of the Ab fragment of the amyloid precursor protein [228,229]. Disruption of nuclear architecture of progenitor cells has been observed [230]. The advent of CRISPR interference technology, which enables flexible targeting of genes upregulated in disease states [231,232,233], can be applied to target and limit abnormal activation of pathways discovered using in vitro models [234].

In an excellent demonstration of organoid technology for disease modeling, a recent study modeled neuronal phenotypes using iPS lines carrying MAPT mutations that underlie frontotemporal dementia [235]. Extensive biochemical, morphological, and transcriptomic characterization identified accelerated neuronal maturation and upregulation of synaptic genes as a key feature associated with MAPT mutations. *ELAVL4*, a splicing factor involved in neuronal maturation, was identified as a core master-regulatory gene upregulated in mutant cells, leading to transcriptome-wide changes in splicing and changes to synaptic recycling rates. This could be reversed by inhibition of PIKFYVE, a lipid kinase that regulates endolysosomal trafficking, consistent with findings in a mouse model of *C9ORF72* hexanucleotide repeat expansion that underlies both frontotemporal dementia and amyotrophic lateral sclerosis [236].

## 10. Evolutionary Insights

The mammalian neocortex is a highly evolved structure, but even across mammals it shows substantial variation in size, cellular organization, and folding pattern [237]. Many hypotheses have been put forward about the developmental mechanisms that might underlie these differences across species [238,239,240,241,242,243,244]. Of particular interest are those changes that might underlie the remarkable adaptations of the human brain to higher cognition, but for practical reasons, many studies have thus far focused on the analysis of progenitor cell proliferation, which might underlie the disproportionate evolutionary expansion of the human cerebral cortex. 

Three prominent hypotheses have been promoted to account for the expansion of the neocortex. One, changes to the proliferative expansion of the neuroepithelial stem cells, which serve as the founder population of cortical radial glia [245]. Two, a reduction in programmed apoptotic cell death of neuroepithelial or radial glial cells [246]. Three, the expansion of the secondary proliferative populations in the outer subventricular zone [54,238,247] is driven by changes in gene expression or activity-dependent processes [248].

Cerebral organoids have the potential to serve as an experimental platform for establishing causality between genetic changes between species and specific neuro-developmental or neurophysiological phenotypes. Two main approaches have emerged so far in the field.

The first approach has been to use organoids to study the function of genetic variants that have been identified as derived from the human lineage [249,250,251,252]. Two examples of variants include NOTCH2NL and NOVA1. NOTCH2NL has been shown to control the production and proliferation of radial glia cells [253,254]. The archaic allele of NOVA1 has been shown to increase progenitor cell apoptosis [255]. Both of these mechanisms are consistent with the prevailing hypotheses about human brain expansion.

The second approach has been to derive brain organoids from multiple species. Thanks to the discovery of somatic reprogramming factors, it is possible to derive brain organoids from many species, including great apes [256,257,258,259]. By comparing organoid differentiations from human and chimpanzee, Mora-Bermudez et al. identified differences in cell cycle kinetics between species [260] and identified candidate modern human-specific causative mutations [261]. Pollen et al. demonstrated that organoids derived from non-human primates recapitulate cross-species gene expression differences observed in primary tissue and identified changes in mTOR signaling pathway activation [32]. Benito-Kwiecinski reported differences in neuroepithelial stem cell proliferation [262]. In addition, Kanton et al. captured transcriptomic and epigenomic differences in developmental trajectories in human and chimpanzee organoids, including changes that overlap with human accelerated regions, and confirmed at least a subset of these molecular differences in primary human and non-human primate prefrontal cortex tissue [263].

Organoids derived from tetrapoid iPS cells generated by fusing cells from different species provide another avenue for interrogating *cis*- and *trans*-regulatory programs for transcriptional divergence between species [264]. As an example, cortical organoids derived from human-chimpanzee tetraploid iPS cells showed accelerated expression of gliogenic programs and increased expression of human somatostatin receptor 2. Differential expression of these pathways between humans and chimpanzees represents yet another candidate pathway that may have contributed to the evolutionary expansion of the human cerebral cortex.

## 11. Conclusions

In the last two decades, pluripotent stem cell-derived brain organoids have advanced from a novel finding to the premier research tool for studying human brain development and disease. These models are utilized by hundreds of researchers. Many challenges remain to improve the fidelity of organoids relative to in vivo tissues and to expand the features of brain development and function that can be effectively studied in these cell culture models. However, there is tremendous enthusiasm to tackle these issues, as outlined in the research reviewed here. In vitro differentiation of human pluripotent stem cells to cerebral organoids offers a tremendous opportunity to understand human neurodevelopment, identify the consequences of disease-relevant mutations, and begin to develop strategies to advance regenerative medicine applications.
